# Efficacy of Behavioral Economic Nudges to Assist Teen Mothers: the Healthy Adolescent Transitions Randomized Controlled Trial

**DOI:** 10.1007/s11121-024-01660-3

**Published:** 2024-03-02

**Authors:** Jack Stevens, Joseph Rausch, Ngozi Osuagwu, Robyn Lutz

**Affiliations:** 1 Nationwide Children’s Hospital, NEOB 3rd Floor, Columbus, OH 43205 USA; 2 Department of Pediatrics, Ohio State University, Columbus, OH USA; 3 OhioHealth Research and Innovation Institute, Columbus, OH USA; 4 Ohio University Heritage College of Osteopathic Medicine, Columbus, OH USA

**Keywords:** Adolescent, Pregnancy, Behavioral economics, Nudges

## Abstract

Communities may often lack the resources to deliver intensive programs to assist teen mothers, and many eligible adolescents may decline participation in lengthy interventions. Therefore, alternative approaches involving less resource and time may be needed. Behavioral economics (BE) can inform the development of such novel interventions. BE often feature low-intensity approaches designed to “nudge” people to help them reach their long-term goals. Nudges can include giving reminders, making the desired behavior more convenient, and optimizing the verbal presentation of recommended options. Three hundred thirty-one American adolescents (ages 14 to 19) who were 22 to 35 weeks pregnant were enrolled in the present trial. One hundred sixty-six participants were randomly assigned to the intervention condition featuring a three-month BE intervention delivered by a registered nurse and social worker. The remaining 165 youths were assigned usual care. Surveys were completed at baseline, 3 months, 12 months, and 18 months. Data collection occurred from 2017 to 2021. Qualitative feedback indicated that the BE intervention was well-received by adolescents. However, there were no significant differences between the intervention and control groups at any time point regarding repeat pregnancy, contraceptive usage, financial literacy, school completion, job attainment, HPV vaccinations, nicotine usage, perception of having a medical home, urgent care/ED usage, and nutritional intake (all *p* > .05). Our findings suggest that a BE-based intervention may not be sufficient to facilitate change for teen mothers. Future programs should consider lasting longer, featuring a higher dose, and/or incorporating systems-level changes. This trial was prospectively registered (NCT03194672 clinicaltrials.gov).

**Supplementary Information:**

The online version contains supplementary material available at 10.1007/s11121-024-01660-3.

## Introduction

According to the National Center for Health Statistics, one out of every six births in 2019 for teenage mothers was a result of a repeat pregnancy (Martin et al., 2021). A review of 30 years of scientific literature regarding repeat teen pregnancy indicated that repeat teen mothers were at heightened risk for adverse outcomes such as unemployment and high school discontinuation relative to first-time teen mothers (Maslowsky et al., 2019). Furthermore, a brief interpregnancy interval (less than 18 months) has been associated with an elevated likelihood of prematurity regarding mothers in general (Conde-Agudelo et al., 2006) and US adolescent mothers in particular (Nerlander et al., 2015).

Perhaps the most well-known program to assist adolescent mothers is the Nurse Family Partnership program. While this intervention has strong empirical support and addresses several domains (e.g., repeat pregnancy, self-sufficiency), its curriculum lasts roughly 2.5 years and recommends dozens of home visits (Nurse Family Partnership, 2021). Communities may often lack the resources to deliver such an intensive program that costs approximately $10,000 per family to deliver (Wu et al., 2017). Furthermore, many teen mothers may decline participation in such a lengthy intervention. A study of attendance patterns for nearly 67,000 mothers from NFP sites across the United States revealed that just 22% consistently participated for the entire duration of the program (Holland et al., 2018). Seventy percent of NFP mothers left the program sooner than recommended. Therefore, alternative approaches involving less resource and time may be needed.

Behavioral economics (BE) can inform the development of such novel interventions. BE is an interdisciplinary framework featuring insights from psychology and economics to facilitate healthy changes (Patel et al., 2018). BE often features low-intensity approaches designed to “nudge” people towards reaching their aspirations. Opportunities are designed in ways to encourage short-term choices that will facilitate completion of long-term goals (e.g., health, financial stability). Nevertheless, individual freedom and choice are preserved. Nudges can include giving reminders, making the desired behavior more convenient, and optimizing the verbal presentation of recommended options (Sunstein, 2016, 2022). Nudges can help overcome knowledge gaps and people’s—particularly adolescents’—natural focus on the short-term consequences of their choices (Sunstein, 2016; Wong et al., 2021). Nudging can have a more favorable benefit–cost tradeoff relative to more resource-intensive interventions (e.g., financial incentive programs, comprehensive educational initiatives) for diverse outcomes (e.g., retirement savings, energy conservation) (Benartzi et al., 2017).

A meta-analysis of over 200 studies concluded that nudges often produce small to moderate improvements across diverse behavioral domains, including health decisions (which included adolescent sleep and vaccinations), food selection, and financial choices (Mertens et al., 2022). Just six percent of those nudges focused on outcomes for children and adolescents. An outside group of experts recently proposed that behavioral economics represents a novel, developmentally sensitive approach to improving adolescent and young adult health (Wong et al., 2021). However, previous interventions have not utilized a BE framework to assist pregnant adolescents across multiple domains. Instead, existing programs (e.g., Nurse Home Visitation, Healthy Families America) have featured more comprehensive education and/or social support lasting a few years (Healthy Families America, 2023).

Through the present randomized trial, a three-month BE program entitled “Healthy Adolescent Transitions (HAT)” was delivered to facilitate goal completion of teenage mothers. The present sample was restricted to adolescents on Medicaid because the HAT intervention was designed to reach youths at greatest risk for psychosocial challenges. Shah et al. (2012) have suggested that the difficulties resulting from scarcity (e.g., low income) interfere substantially with short-term decisions, which in turn impact long-term goal attainment. Through experiments regarding financial choices, Mrkva et al. (2021) found that “low-SES [socioeconomic status] consumers were impacted more by nudges, meaning nudges that facilitated the selection of a good option benefited them more than high-SES individuals” (pg. 80).

In addition to pregnancy prevention, other domains—such as financial literacy, educational/career attainment, and healthy life skills (e.g., promoting smoking cessation, increasing human papillomavirus (HPV) vaccinations, establishing a medical home, and improving nutrition)—are important for this adolescent population. The HAT program addressed these secondary domains due to their substantial impact on health and well-being.

Relative to participants from a usual care control condition, our primary hypothesis was that adolescent mothers receiving the HAT intervention would have lower rates of repeat pregnancy. Our secondary hypotheses were that HAT versus usual care participants would have lower nicotine use as well has higher rates of the following: contraceptive usage, financial literacy, school completion, job attainment, HPV vaccination, medical home utilization, and healthy nutrition. Our exploratory aims were to examine differences in optimal birth control coverage (immediate post-partum placement of long-acting reversible contraception) and overall life satisfaction for HAT versus control participants. Finally, we examined qualitative impressions of the HAT program to assess participant perspectives of the strengths and limitations of this novel initiative.

## Methods

### Participants

Three hundred thirty-one adolescents were enrolled in the present trial. Baseline eligibility criteria were (1) 14–19 years old; (2) English speaking; (3) internet and phone access; (4) pregnant (22–35 weeks gestation); and (5) having Medicaid coverage.

From July 2017 to May 2019, study participants were recruited during outpatient appointments at clinics providing prenatal services. All clinics were associated with a large regional Midwestern health system. Roughly 90% of the participants were recruited through clinics located in a large metropolitan area. The remaining 10% were recruited through clinics located in small cities. Interested and willing youths 18 or 19 years old provided informed written consent for study participation. A guardian provided informed written consent for the younger participants. Participants under 18 years of age provided informed written assent for study participation.

### Procedures

A randomized controlled trial of the HAT intervention was conducted involving individual-level assignment that occurred after a baseline survey was completed. Each participant had a 50% chance of being randomly assigned to the HAT intervention condition, and a 50% chance of being randomly assigned to the Usual Care control condition. Prior to the start of the trial, an administrative assistant filled opaque envelopes one at a time containing condition assignment (intervention or control) based upon statistician-generated random number sequences. Block sizes randomly varied over time from 4 (2 intervention, 2 control) to 6 (3 intervention, 3 control).

We stratified the random assignment by baseline age (18 or 19 years versus under 18 years). We were concerned that baseline age differences could have influenced our findings. On the one hand, older adolescents might have greater privacy and independence to receive contraceptive services. On the other hand, older adolescents historically have higher teenage pregnancy rates relative to younger adolescents. We also stratified the random assignment by recruitment site because standard-of-care may have differed across clinics. One hundred sixty-six adolescents were assigned to the intervention condition, and the remaining 165 youth were assigned to the control condition.

There were four time points for our study surveys—baseline, 3 months post-enrollment (immediate post-intervention), 12 months post-enrollment (short-term follow-up, occurring 9 months after the intervention ended), and 18 months post-enrollment (long-term follow-up, occurring 15 months after the intervention ended). Regarding the timing for the last survey, the long-term follow-up period was changed from 21 to 18 months for all study participants. This modification from the originally registered study procedures allowed for three extra months of participant recruitment.

The primary and secondary outcomes assessed by the surveys are described in Table 1. Three of the outcomes were assessed based upon items from previous materials. Regarding the primary outcome of rapid repeat pregnancy prevention, past research indicated that such a self-report item was valid when compared against this administrative data source: state-level birth certificate files (Stevens et al., 2019). Regarding financial literacy, the five items were utilized based upon the Money Matters Curriculum (The Gallup Organization, 2006). Regarding nutritional intake, the items were taken from the Youth Risk Behavioral Survey (Centers for Disease Control & Prevention, 2015).

**Table 1:** Description of primary and secondary outcome measures

Outcome	Description
Repeat pregnancy	An additional pregnancy after index pregnancy at baseline – primary outcome
Use of long-acting	Any use of an IUD or an implant within the last 3 months
Reversible	
Contraception (LARC)	
Use of any contraception	Any use of any form of birth control within the last 3 months
Financial literacy	Knowledge scores to five true–false questions about basic topics, including banks, budgeting, credit cards, and check-cashing stores
School completion	Completion of more schooling relative to baseline education level
Job attainment	Any paid work outside of the home in the last 3 months
HPV vaccinations	Total number of HPV shots received in a lifetime
Nicotine usage	Any cigarette/vaping product use in the last 3 months
Medical home	Perception of having a regular primary care provider
Use of emergency/Urgent Care	Number of visits for these services in the last 6 months
Nutritional intake	Number of times a food/beverage item was consumed in the last 7 days (on 7 point Likert scale from none to 4 + times per day)

**Fig. 1:**
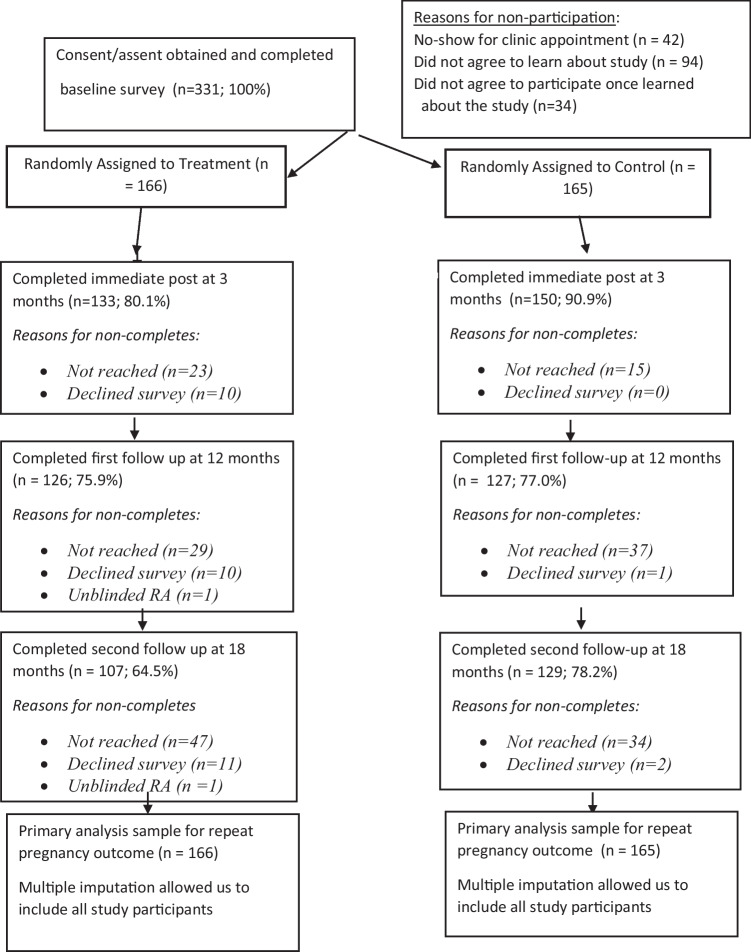
CONSORT diagram

A blinded research assistant administered outcome surveys by telephone. That bachelor’s level study team member was from an independent entity in charge of the evaluation that was separate from the organizations involved in delivering the HAT intervention. The research assistant had a 3-month window to complete each of those surveys. In other words, 3-month surveys were due roughly 90 to 180 days post-enrollment, 12-month surveys were due roughly 360 to 450 days post-enrollment, and 18-month surveys were due roughly 540 to 630 days post-enrollment. Figure 1 features the CONSORT flow diagram.

Multiple steps were taken to ensure that the research assistants were blinded to a particular adolescent’s study condition. First, the research assistants were not present during the HAT providers’ openings of opaque envelopes that revealed a particular adolescent’s study condition. Second, HAT providers conducted home visits and phone calls in closed rooms where the research assistants were not located. Third, case discussions regarding progress and challenges for individual HAT recipients occurred without the research assistants present.

Participants in both study conditions received $10, $20, $30, and $40 gift cards for completing the baseline, 3-month, 12-month, and 18-month surveys respectively. At the beginning of the study, the institutional review board (IRB) from the local evaluator entered into a reliance agreement with the IRB from the lead hospital that delivered the intervention condition. All study procedures were approved by the lead entity’s IRB. The trial (NCT03194672) was registered at clinicaltrials.gov.

### Study Conditions

#### Intervention Condition

The Healthy Adolescent Transitions (HAT) intervention was a 3-month program with an intended dose of 12 weekly sessions lasting approximately 60–90 min each. The program was delivered by a multidisciplinary team of two providers—a registered nurse and a bachelor’s level social worker—who met with an individual adolescent. Depending upon the content of a particular session, sometimes only a nurse would participate, sometimes only a social worker would participate, and sometimes both professionals would interact simultaneously with the adolescent.

During the first interaction with the adolescent, the nurse/social worker team would provide a brief overview of the HAT content areas. The curriculum was flexible for each adolescent. In other words, the adolescent would be allowed to select which content area(s) she wished to discuss further. We thought this flexibility was consistent with the “freedom-preserving” notion of nudges—a phrase from one of the leading behavioral economic experts (Sunstein, 2016). Furthermore, we were concerned that a fixed curriculum might discourage teens from continuing participation if they were not interested in certain topics. The adolescent would choose how often she wished to interact with the nurse and/or social worker.

Each content area and the BE strategies for each of those domains is described briefly in Table 2. The HAT intervention was based on the nudges described in that table. Six health care providers—three registered nurses, two social workers, and an obstetrician/gynecologist—with past clinical experience with adolescents became educated about nudging and selected the strategies they viewed as most appropriate for each content area. A doctoral-level psychologist with expertise in behavioral economics verified the appropriateness of their selections. Fidelity checklists were then created to guide the nurse/social worker team in addressing each topic selected by an adolescent. Across the course of the study, the program director observed a handful of in-person substantive encounters and verified that BE strategies documented through intervention notes by HAT providers were the strategies actually delivered.

**Table 2:** Overview of behavioral economic (BE) strategies for each content area

Content area	BE strategy^a^	Description
Contraception^b^	“Structuring choices”	Highlight advantages of long-acting reversible contraception, including high efficacy levels and no requirement of frequent maintenance behaviors
	“Ordering effects”	First presenting long-acting reversible contraception before presenting the other methods
	“Using visual effects”	Encourage placement of bright, visual reminders in hospital overnight bags to prompt adolescents to initiate birth control discussions during labor and delivery stay
Financial literacy	“Structuring choice”	Discuss benefits of establishing a local bank account and budgets through the Money Matters curriculum
School/job attainment	“Simplifying and easing of current requirements”	Aid with breaking educational goals into tasks, resume building, interviewing skills, professional attire attainment
HPV vaccinations	“Providing reminders”	Assist with setting automated prompts to seek these vaccinations once medically eligible post-pregnancy
Nicotine usage	“Enlisting loss aversion”	Present charts detailing the considerable expenses of cigarette smoking
	“Structuring choices”	Present charts detailing the long-term, as opposed to daily, costs of nicotine use
Establishing a medical home	“Simplifying and easing of current requirements”	Provided transportation to two primary care appointments through study van
	“Making contexts or policies easily navigable”	Have adolescents use location websites to find primary care providers in their locale/zip code
Nutritional intake^c^	“Simplifying and easing of current requirement”	Distribute free kitchen supplies and small, one-time samples of healthy food
	“Using visual effects”	Encourage placement of healthy foods in prominent places (e.g., fruit bowl on kitchen table or counter) and offering transparent containers for refrigerator storage of fruits/vegetables
	“Increasing fun”	Encourage consumption of nonpreferred vegetables (e.g., spinach) through fruit smoothies

^a^Based on Sunstein list of 31 “Freedom-Preserving Tools or ‘Nudges’” (Sunstein, 2016, pg. 718]

^b^A board-certified obstetrician/gynecologist provided training and ongoing consultation to the HAT nurses to ensure that medically accurate information (e.g., efficacy, side effects, reversibility) about contraception was conveyed to the intervention participants

^c^A doctoral-level behavioral economist with specific expertise in promoting better nutrition advised the HAT providers on strategies to promote health eating

One hundred thirty-two out of 166 intervention participants had at least one substantive encounter with a HAT provider. An encounter was considered substantive if content was covered, as opposed to those interactions that solely involved scheduling. Each content area was discussed with 61% to 80% of those intervention group participants with at least one substantive encounter. Sixty-nine percent of the intervention group participants with at least one substantive encounter discussed birth control, the topic proximally related to the primary outcome of pregnancy prevention. On average, each content area was discussed over 1 to 3 substantive encounters if that topic was selected by a participant. Supplemental Table 1 features further details about the topics selected by participants. Two-thirds of the encounters featured both a nurse and a social worker interacting simultaneously with an adolescent. Ninety-one intervention group participants out of 166 total intervention group members (54.5%) received free kitchen supplies from the HAT providers to facilitate healthy eating.

Participants had 5.5 substantive encounters on average. Twelve participants (9.1% of those with at least one substantive encounter) had 12 or more encounters—the originally intended dose. Thirty-two participants (24.3%), 37 participants (28.0%), and 51 participants (38.6%) had 7–11, 4–6, and 1–3 substantive encounters respectively. Substantive encounters occurred 63.5% of the time in the participant’s home, 6.7% of the time by telephone, and 29.6% of the time in another setting (e.g., community setting such as a private area of a library or restaurant). Total duration across all substantive encounters lasted nearly 5 h per participant (Mean = 295 min, SD = 241 min). There were no differences by baseline age (18 or 19 years versus younger) regarding total duration (*p* = .65).

#### Control Condition

All participants in the intervention and the control conditions received a welcome older after they signed the consent/assent forms and before they were randomized to study condition. The folder contained five brochures on birth control and sexually transmitted infections.

In addition, similar to intervention participants, control participants were eligible to receive standard-of-care health and social services in their area. For example, all participants were eligible to receive care from prenatal clinics and inpatient labor/delivery. However, control participants had no interactions with the HAT providers. The control participants were ineligible to receive HAT services for the entire duration of the randomized trial, not just the 3-month post-enrollment period in which intervention participants could receive the HAT curriculum.

After all of the outcome questions were administered from the final (18 month) survey, all participants were asked if they received any programming outside of the study during the previous 2 years. The intervention and control groups did not differ regarding percentage who reported receiving such usual care programming (48.6% versus 38.8%; chi-square = 2.307, *p* = .13).

### Analytic Plan

#### Overview of Our Statistical Approaches

Our primary, secondary, and exploratory research questions were conducted using an intent-to-treat approach. We define intent-to-treat as estimating the impact of being randomly assigned to intervention versus control group, regardless of how many (if any) HAT encounters each intervention group participant actually received. We used one benchmark approach and three sensitivity approaches to examine the primary, secondary, and exploratory outcomes.

#### Benchmark Analysis

We used logistic or linear regression models, depending on whether the outcome was dichotomous or continuous, respectively. We used multiple imputation to account for missing data. This approach was carried out in two stages. First, a set number of imputed data sets were generated with auxiliary variables used to facilitate prediction of the missing data. Then, there was an analysis of each of the imputed data sets with the model of interest, and the results were aggregated. We imputed 50 datasets, separately for treatment and control conditions (Jakobsen et al., 2017; Sullivan et al., 2018). The fully conditional specification method was used for imputation based on linear regression for continuous variables and generalized logistic regression for categorical variables using the MI procedure in SAS 9.4. Imputation model results were then analyzed with the appropriate analysis model and summarized using the MIANALYZE procedure in SAS 9.4. The imputation model included the covariates used within the analysis model (i.e., recruitment site, age, race), the outcome variable of interest at the specified time point, and the same outcome variable measured at other time points (when available). The analysis model included the outcome variable at a given post-intervention time point as the analytic outcome of interest, condition (intervention vs control) as the primary predictor of interest, and the baseline measure of the outcome variable (when applicable), race, age, and site as covariates.

Sensitivity Analysis 1: A complete case (i.e., listwise deletion) approach was used to analyze the outcome of interest with linear or logistic regression, depending on whether the outcome was categorical or continuous, with condition and the same covariates as those used in the benchmark approach included in the analysis. This “complete” case approach reflects no missing survey responses.

Sensitivity Analysis 2: This approach was the same as the benchmark approach except that all baseline variables that were predictive of missingness at one of the time points at the *p* < .10 level were included in the imputation model only. Thus, the analysis model was the same as the benchmark model. If the model did not run using the *p* < .10 criterion, the set of variables was reduced by using the *p* < .05 criterion for including variables in the imputation model.

Sensitivity Analysis 3: For the continuous outcomes, a repeated measures model was used based on restricted maximum likelihood estimation, was used for the quantitative outcomes using the MIXED procedure in SAS 9.4. The Kenward-Rogers approach to denominator degrees of freedom and standard errors was used to obtain more accurate statistical inference.

Power Analysis. Assuming 20% attrition, an alpha = 0.05, and power = 0.80, we were powered to detect an approximately 40% decline in repeat pregnancy rates in the HAT intervention group relative to the control group based upon the initial planned sample size of 500. Specifically, if the repeat pregnancy rates of the control group were either 25%, 30%, or 35%, we could have detected significant differences if the HAT intervention group had rapid repeat pregnancy rates of 14%, 18%, and 22%, respectively. Those originally hypothesized rates of repeat pregnancy in the control and intervention groups at 18 months were based upon past research of two empirically-supported pregnancy prevention programs—Nurse Family Partnership (Olds et al., 2002) and Teen Options to Prevent Pregnancy (Stevens et al., 2017). With our actual sample size of 331, we had 80% power to detect an approximately 13% absolute decline in repeat pregnancy rates in the intervention group relative to the control group.

## Results

### Baseline Characteristics

Table 3 presents summary statistics regarding key demographic and outcome values at baseline. Roughly ten percent of the sample was Hispanic, a third of the sample was Non-Hispanic Black, and half of the sample was Non-Hispanic White. Roughly half of the sample had completed at least the 12th grade or a GED. *T*-tests and chi-square tests revealed that the intervention and control groups did not differ at baseline on any demographic or outcome variable (all *p*-values > .20).

**Table 3:** Baseline values of key demographic and outcome variables

**Baseline measure**	**Intervention proportion or mean (standard deviation)**	**Control proportion or mean (standard deviation)**	**Intervention versus control difference**	**Intervention versus control *p*-value of difference**
Age (166/165)	18.1 (1.1)	17.9 (1.3)	0.2	.26
Race/ethnicity (165/165)			Chi-square(3) = 1.59	.66
Hispanic	0.08	0.10		
Non-Hispanic White	0.47	0.52		
Non-Hispanic Black	0.34	0.29		
Other	0.10	0.08		
Any LARC usage in the 3 months before current pregnancy (162/162)	0.07	0.09	-0.02	.68
Any contraceptive use during 3 months before current pregnancy (165/165)	0.63	0.61	0.02	.73
Financial Literacy Score (166/165)	2.93 (1.3)	2.78 (1.3)	0.15	.25
Completed at least 12th grade/GED (164/163)	0.51	0.48	0.03	.70
Any amount of paid work hours (166/164)	0.41	0.45	-0.04	.51
Number of HPV shots received (77/78)	1.2 (1.3)	1.2 (1.3)	0.0	.85
Uses nicotine (166/165)	0.20	0.20	0.00	.98
Perceives having a medical home (165/165)	0.41	0.39	0.02	.65
Interest in healthy eating (165/165)	3.55 (0.7)	3.42 (0.8)	0.13	.12
Interest in getting more schooling (165/165)	3.24 (1.0)	3.12 (1.1)	0.12	.32
Interest in becoming employed outside the home (165/163)	3.58 (0.8)	3.45 (0.9)	0.13	.18
Trying to avoid getting pregnant over next year (165/164)	72.7	68.9	3.8	.67

LARC refers to long acting reversible contraception. The numbers in parentheses that are immediately after the name of each baseline measure refer to sample size of intervention and control participants completing each measure. Interest in healthy eating, getting more schooling, and becoming employed outside of the home was based on a 1 (“not at all interested”) to 4 (“very interested”) scale. The last variable had four possible responses: “trying to get pregnancy,” “neither trying to get pregnant nor trying to avoid getting pregnant,” “trying to avoid getting pregnant,” and “don’t know”

### Primary Outcome

Table 4 presents the results regarding our primary and secondary hypotheses. Our primary hypothesis was that adolescent mothers receiving the HAT intervention would have lower rates of repeat pregnancy. The intervention and control groups did not differ regarding adolescent report of a repeat pregnancy occurring within the 18-month post-enrollment period (24% versus 30%; *p* = .27). As part of an exploratory analysis, we investigated if there were group differences in reporting a repeat pregnancy through the 12- and/or 18-month surveys. The difference between the intervention and control group was still not significant (24% versus 31%, *p* = .14). For intervention participants, there was no difference regarding number of sessions in which contraception was discussed regarding those who did versus did not report a repeat pregnant at 18 months (*p* = .38).

**Table 4:** Benchmark outcome analyses estimating group (intervention versus control) differences

**Outcome measure**	**Intervention proportion or mean (SD)**	**Control proportion or mean (SD)**	**Differences (*p*-values of differences)**
Primary outcome			
Repeat pregnancy at 18 months (107/129)	0.24	0.30	aOR = 0.66 (*p* = .27)
Contraception			
Any LARC usage at 12 months (126/127)	0.40	0.34	aOR = 1.30 (*p* = .33)
Any LARC usage at 18 months (107/129)	0.36	0.32	aOR = 1.24 (*p* = .48)
Any contraceptive usage at 12 months (126/127)	0.78	0.84	aOR = 0.67 (*p* = .25)
Any contraceptive usage at 18 months (107/129)	0.76	0.79	aOR = 0.79 (*p* = .48)
Financial literacy			
Financial literacy at 3 months (133/150)	3.39 (1.19)	3.20 (1.19)	0.15 (*p* = .28)
Financial literacy at 12 months (126/127)	3.51 (1.15)	3.26 (1.24)	0.17 (*p* = .20)
Financial literacy at 18 months (107/129)	3.58 (1.16)	3.35 (1.03)	0.17 (*p* = .20)
Educational attainment			
Higher school completion at 3 months (131/148)	0.18	0.09	aOR = 2.34 (*p* = .13)
Higher school completion at 12 months (124/124)	0.33	0.24	aOR = 2.11 (*p* = .10)
Higher school completion at 18 months (105/127)	0.32	0.26	aOR = 1.82 (*p* = .16)
Job attainment			
Working at 3 months (133/150)	0.44	0.36	aOR = 1.75 (*p* = .07)
Working at 12 months (126/127)	0.54	0.61	aOR = 0.63 (*p* = .11)
Working at 18 months (107/127)	0.66	0.59	aOR = 1.47 (*p* = .22)
HPV vaccinations			
Number of HPV shots at 12 months (63/52)^a,b^	2.21 (1.20)	2.14 (1.14)	0.02 (*p* = .92)
Number of HPV shots at 18 months (60/50)^a,b^	2.31 (1.13)	1.86 (1.30)	0.41 (*p* = .075)
Nicotine usage			
Nicotine use at 3 months (133/150)	0.17	0.20	aOR = 0.68 (*p* = .43)
Nicotine use at 12 months (126/127)	0.28	0.33	aOR = 0.68 (*p* = .29)
Nicotine use at 18 months (107/129)	0.28	0.33	aOR = 0.77 (*p* = .49)
Medical home			
Perceive having a medical home at 3 months (133/150)	0.42	0.34	aOR = 1.35 (*p* = .26)
Perceive having a medical home at 12 months (126/127)	0.43	0.38	aOR = 1.35 (*p* = .26)
Perceive having a medical home at 18 months (107/128)	0.50	0.43	aOR = 1.48 (*p* = .17)
Urgent care/ED usage at 3 months (133/149)	1.04 (1.84)	0.89 (1.54)	0.15 (*p* = .45)
Urgent care/ED usage at 12 months (126/127)	1.27 (2.43)	0.97 (1.17)	0.30 (*p* = .24)
Urgent care/ED usage at 18 months (107/128)	1.29 (4.05)	1.11 (1.40)	0.25 (*p* = .55)
Nutritional intake			
100% Fruit juice consumption at 3 months (133/150)	4.55 (5.72)	5.34 (7.54)	−0.88 (*p* = .28)
100% Fruit juice consumption at 12 months (126/127)	4.57 (5.82)	5.13 (6.75)	−0.47 (*p* = .57)
100% Fruit juice consumption at 18 months (107/129)	5.63 (7.52)	4.81 (6.49)	0.83 (*p* = .35)
Fruit consumption at 3 months (133/150)	6.15 (5.97)	5.73 (5.78)	0.46 (*p* = .52)
Fruit consumption at 12 months (126/127)	5.14 (5.93)	3.98 (4.69)	1.17 (*p* = .08)
Fruit consumption at 18 months (107/129)	5.21 (6.10)	4.81 (4.50)	0.43 (*p* = .52)
Salad consumption at 3 months (133/150)	1.63 (2.40)	1.81 (2.57)	−0.09 (*p* = .76)
Salad consumption at 12 months (126/127)	1.71 (2.64)	1.81 (2.49)	−0.11 (*p* = .75)
Salad consumption at 18 months (107/129)	1.37 (1.96)	1.80 (2.42)	−0.40 (*p* = .14)
Potato (non-fried) consumption at 3 months (133/150)	1.90 (2.41)	2.59 (3.75)	−0.67 (*p* = .072)
Potato (non-fried) consumption at 12 months (126/127)	2.33 (2.50)	2.63 (3.76)	−0.35 (*p* = .36)
Potato (non-fried) consumption at 18 months (107/129)	2.38 (3.06)	2.30 (2.19)	0.08 (*p* = .81)
Carrot consumption at 3 months (133/150)	1.01 (1.59)	1.39 (3.83)	−0.30 (*p* = .39)
Carrot consumption at 12 months (126/127)	0.97 (1.87)	1.27 (2.79)	−0.28 (*p* = .34)
Carrot consumption at 18 months (107/129)	0.91 (1.69)	0.90 (1.46)	0.003 (*p* = .99)
Other vegetable consumption at 3 months (133/150)	3.66 (3.42)	4.21 (4.49)	−0.49 (*p* = .32)
Other vegetable consumption at 12 months (126/127)	3.17 (3.97)	3.53 (4.33)	−0.27 (*p* = .58)
Other vegetable consumption at 18 months (107/129)	3.37 (3.77)	3.64 (4.02)	−0.23 (*p* = .62)
Milk consumption at 3 months (133/150)	6.00 (6.88)	5.27 (6.55)	0.70 (*p* = .38)
Milk consumption at 12 months (126/127)	3.58 (4.95)	3.26 (5.03)	0.42 (*p* = .50)
Milk consumption at 18 months (107/129)	4.56 (6.84)	3.70 (5.22)	0.90 (*p* = .25)
Breakfast consumption at 3 months (132/150)	4.59 (2.67)	5.18 (2.46)	−0.50 (*p* = .088)
Breakfast consumption at 12 months (126/127)	3.98 (2.75)	4.20 (2.84)	−0.12 (*p* = .74)
Breakfast consumption at 18 months (107/128)	3.95 (2.86)	4.28 (2.65)	−0.27 (*p* = .45)
Soda consumption at 3 months^c^ (133/150)	18.01 (9.21)	17.60 (8.98)	1.03 (*p* = .30)
Soda consumption at 12 months^c^ (126/127)	15.99 (9.54)	16.10 (9.70)	0.29 (*p* = .79)
Soda consumption at 18 months^c^ (107/129)	17.07 (9.81)	18.96 (9.15)	−1.44 (*p* = .21)

LARC refers to long-acting reversible contraception. The numbers in parentheses that are immediately after the name of each outcome measure refer to sample size of intervention and control participants completing each measure. Proportions/means are the estimates obtained across the imputed data sets. Differences and *p*-values of differences refer to results from regression analyses. aOR refers to adjusted odds ratios

^a^These sample sizes were much lower than the other sample sizes for other outcomes, because many adolescents reported being unaware of how many HPV shots they received

^b^Due to algorithmic issues, the imputation model for number of HPV shots excluded site, although this covariate was still included in the analysis model

^c^To be consistent with the other nutrition items, soda consumption was reversed score so that higher scores reflected better nutrition. The observed values correspond to adolescents averaging a few sodas per week. Diet sodas did not count as sodas. It should also be noted that nutritional intake items were analyses separately because attempts to combine items into internally consistent subscales were unsuccessful

### Secondary Outcomes

Our secondary hypotheses were that HAT versus usual care participants would have lower nicotine use as well has higher rates of the following: contraceptive usage, financial literacy, school completion, job attainment, HPV vaccination, medical home utilization, and healthy nutrition. There were no significant differences between the intervention and control groups at any time point regarding any of these outcomes (all *p* > .05).

### Exploratory Outcomes

We investigated differences in life satisfaction scores at 3, 12, and 18 months based upon a 4-point scale ranging from mostly dissatisfied to mostly satisfied. There were no group differences at any time point (*p*-values ranged from .39 to .52).

In addition, we investigated if the groups differed regarding immediate post-partum placement of long-acting reversible contraception (LARC) according to the 12-month survey. The intervention and control groups did not differ on this exploratory variable (18% versus 21%, *p* = .73). Immediate post-partum placement of long-acting reversible contraception helps ensure the most timely form of birth control coverage.

### Sensitivity Analyses

All 52 outcomes listed in Table 4 were examined using each of the first two sensitivity approaches; only the 35 continuous outcomes were examined using the third sensitivity approach. Just one significant difference emerged out of 139 (52 + 52 + 35) total comparisons. When variables predicting of missingness were in the imputation model, intervention group participants were more likely to be working at 3 months relative to control group participants (*p* = .04).

### Non-response Analysis Regarding 3, 12, and 18 Month Surveys

Out of 33 comparisons, we detected just one significant interaction between survey completion and study condition. Participants from the control condition who did not complete the 18-month survey tended to be younger relative to participants who completed the 18 survey and control participants who did complete the 18-month survey (*p* = .041).

### Qualitative Responses

Supplemental Table 2 outlines data collection and coding procedures for this feedback. A convenience sample of forty-nine of the intervention group participants completed this assessment. Forty of those adolescents (82%) reported liking something about the HAT program. Some responses were general (e.g., “It was helpful.”), while other responses specifically highlighted the HAT provider and different content areas (e.g., “It taught me a lot I didn’t know about what is healthy to eat.”). Thirty-nine of those adolescents (80%) indicated that they would change nothing about the HAT intervention. The most common response among the remaining ten adolescents was a request to increase the duration of the program (e.g., “spend more time with you people [the nurse/social worker]”).

No unanticipated adverse outcomes resulting from HAT intervention were reported.

## Discussion

The present investigation featured the first randomized trial of a behavioral economics intervention to assist teen mothers across several different domains. Qualitative impressions from a subset of participants suggest that the HAT intervention was well-received by adolescents. However, we found no significant differences between the intervention and control groups on any primary, secondary, or exploratory outcome utilizing our benchmark statistical approach. This disappointing pattern emerged despite a high risk of false positive results; a total of nearly 60 different outcomes were assessed without a statistical correction for this large number of comparisons. A similar pattern of nonsignificant differences was observed based upon three different sensitivity approaches.

There were multiple methodological strengths of the present study. To begin with, the investigation featured a large and racially/ethnically diverse sample of participants at elevated risk for adverse health and psychosocial outcomes. Furthermore, the post-intervention, short-term follow-up, and long-term follow-up surveys were administered by independent evaluators unaware of a particular adolescent’s study condition. This blinding minimizes bias in assessing the outcomes of an intervention.

However, several limitations deserve mention. To begin with, there was differential attrition at 3 and 18 months. Survey completion rates were lower for the intervention group, compared to the control group, at both of those time points by at least 10 percentage points. Such differential attrition reduces confidence that the intervention and control groups were similar at baseline on all unmeasured characteristics. However, we detected just one significant interaction between survey completion and study condition regarding baseline characteristics. That single interaction was in regard to age, which was a covariate in all of our outcome analyses. This pattern of findings should alleviate, but not eliminate, concerns that differential attrition led to an inaccurate evaluation of the HAT program’s impact. Furthermore, a similar pattern of non-significant group differences on all outcomes emerged across all three time points, including at 12 months when there was minimal differential attrition.

In addition, much of the 18-month outcome data were collected during the first year of the COVID-19 pandemic. This timing may have affected the results regarding rapid repeat pregnancy. On the one hand, stay-at-home orders and social distancing may have reduced opportunities for sexual activities across both study conditions. On the other hand, both groups of participants may have experienced reduced access to contraceptive services, which could have led to increased repeat pregnancies in both groups. Similarly, the pandemic may have limited opportunities for other secondary outcomes (e.g., job attainment, establishment of medical homes). Therefore, the results of the current study may not generalize to non-pandemic time periods.

Finally, while we obtained a large sample of 331 participants, we did not reach our initial recruitment goal of 500 pregnant adolescents. The recruitment challenges may have reflected the national decline in adolescent pregnancy rates over the past several years. The present study may have been underpowered to detect group differences.

Here are three potential explanations for the null findings. First, the duration of the HAT intervention may have been insufficient. Empirically supported repeat pregnancy prevention programs, such as Nurse Family Partnership (Nurse Family Partnership, 2021) and Teen Options to Prevent Pregnancy (Stevens et al., 2017) last from 18 to 30 months. In contrast, the HAT intervention lasted only 3 months and focused largely on the prenatal period. Participants might have been more receptive to certain topics, such as obtaining contraception or seeking employment, a few months after delivery. Participants also may have been more receptive to certain strategies if adolescents—relative to professionals—had a greater role in creating the nudges.

Second, the dose of the HAT intervention may have been inadequate to effect change. Future interventionists should consider using a fixed curriculum in which all content areas are covered in detail while emphasizing that teens may wish to make changes in only some behavioral domains. While the HAT program was initially designed to offer 12–18 h of substantive encounters, the HAT providers actually delivered content across seven possible domains in just five hours on average for each adolescent. Financial literacy was one of the secondary goals for the HAT intervention; previous research suggests that dozens of hours of focused instruction may be needed for even modest impacts in this domain (Ferandes et al., 2014). Similarly, educational attainment was a secondary goal of the HAT intervention; past research indicated that 10–15 h of individual coaching focuses solely on college enrollment were necessary to increase enrollment in post-secondary education (Barr & Castleman, 2016). Smoking cessation was another secondary goal for HAT. Nurses’ highlighting to adolescent mothers the long-term costs of nicotine usage may be insufficient to change this health behavior. Instead, intermediate to long-term pharmacotherapy (e.g., nicotine replacement) may have been more impactful (Claire et al., 2020).

Third, system-level changes—as opposed to an individually focused intervention—may have been necessary to produce desired results in many domains addressed by the HAT nurses and social workers. To begin with, hospital-wide implementation of standing orders to offer all adolescent mothers immediate post-partum placement of long-acting reversible contraception during labor and delivery stays might better prevent rapid repeat pregnancies. Only one-fifth of the present sample reported receiving this timely and effective contraceptive option. In addition, health systems may need to broaden opportunities (e.g., number of clinicians, convenience of locations) for primary care services to low-income pregnant adolescents to facilitate the establishment of medical homes. Finally, locales may need to increase the number of grocers, food banks, and community gardens for low-income families to obtain affordable fruits and vegetables.

## Conclusions

A novel program featuring behavioral economics strategies was not efficacious for adolescent mothers. We speculate that interventions that last longer, feature a higher dose, include other key members of a teen’s social network (e.g., sexual partner), and/or incorporate systems-level changes might deserve future empirical attention to facilitate goal attainment for this population.

### Supplementary Information

Below is the link to the electronic supplementary material.

Supplementary file1 (DOCX 14 KB)

## Data Availability

The declaration statement regarding protocol availability will help readers contact the authors if needed.

## Declarations

### Protocol Availability

The protocol detailing the HAT intervention and outcome measurement is available from the authors upon request.
