# Supplementary material for: Efficacy of Behavioral Economic Nudges to Assist Teen Mothers: the Healthy Adolescent Transitions Randomized Controlled Trial
Source: Prev Sci. 2024 Mar 2;25(3):509–20. doi: 10.1007/s11121-024-01660-3 (PMC11093778; doi:10.1007/s11121-024-01660-3)
Supplement: Supplementary file 1 — Supplementary file1 (DOCX 14 KB) [file 11121_2024_1660_MOESM1_ESM.docx]

**Supplemental Table 1 – Content Received by HAT Participants**

Percentage selecting Average number (SD) of encounters

Content area to discuss this topic in which selected topic was discussed

Contraception 68.9% 2.1 (1.1)

Long-acting 66.7% 1.9 (1.1)

reversible

contraception

Financial literacy 61.4% 1.7 (1.1)

School attainment 68.2% 2.2 (1.5)

Job attainment 59.8% 2.1 (1.1)

HPV vaccinations 63.6% 1.6 (0.9)

Nicotine usage 61.4% 1.7 (1.1)

Medical home 72.0% 2.2 (1.4)

Nutritional intake 80.3% 2.7 (1.9)

Note. Table refer to HAT intervention participants with at least one substantive encounter (n = 132). More than one content area could be discussed during a particular substantive encounter. Financial literacy and nicotine usage were discussed during the same substantive encounters.

**Supplemental Table 2 – Description of Qualitative Data Collection and Coding Procedures**

Step 1 (Data Collection): Participants were asked by telephone: “What did you like best about the HAT program?” and “If you could change something about the [HAT] program, what would it be?” This assessor was neither a HAT provider nor a research assistant administering the outcome surveys. The assessor recorded the responses verbatim.

Step 2 (Coding Procedures): After all qualitative surveys were completed, coding of the responses was then completed by a member of the independent evaluation team who was not aware of that individual respondent’s answers to the 3, 12, and 18-month outcome surveys. Only responses that reflected the content or process of the HAT intervention were coded as answering affirmatively to each of those two questions. Responses that reflected other aspects of the study (e.g., the 3, 12, and 18 month outcome surveys, compensation for completing the outcome surveys) were not coded as answering affirmatively to those two questions.
